# Methicillin-Resistant Staphylococcus aureus (MRSA) Empyema Post-COVID Infection Causing Severe Septic Shock and Multiorgan Failure

**DOI:** 10.7759/cureus.41054

**Published:** 2023-06-27

**Authors:** Antony Arumairaj, Ali Safavi, Hossam Amin, Armeen Poor, Natoushka Trenard

**Affiliations:** 1 Internal Medicine, Metropolitan Hospital Center/New York Medical College, New York City, USA; 2 Surgery, Division of Thoracic Surgery, Metropolitan Hospital Center/New York Medical College, New York City, USA; 3 Internal Medicine/Division of Pulmonary Critical Care Medicine, Metropolitan Hospital Center/New York Medical College, New York City, USA

**Keywords:** multiorgan failure, septic shock, ards, covid 19, multifocal pneumonia, empyema, mrsa

## Abstract

Secondary bacterial infections post-COVID infection posed a major challenge to the healthcare settings during the COVID pandemic. We present the case of an 81-year-old patient who was initially admitted for COVID pneumonia in a tertiary care hospital and was managed with a course of dexamethasone and had a good outcome. However, post-discharge, the patient developed symptoms of productive cough, hemoptysis and shortness of breath. Evaluation of the patient revealed that the patient had developed a secondary bacterial infection with Methicillin-resistant *Staphylococcus aureus* (MRSA), resulting in MRSA pneumonia and empyema. The patient was started on appropriate antibiotics and underwent thoracocentesis followed by video-assisted thoracoscopic surgery (VATS) and decortication. MRSA infection resulted in severe septic shock, acute respiratory distress syndrome (ARDS) and multiorgan failure. Successful intensive care unit (ICU) management of the life-threatening complications resulted in the remarkable recovery of the patient.

## Introduction

The COVID-19 infection resulting from the severe acute respiratory syndrome coronavirus 2 (SARS-CoV-2) virus, which started in the city of Wuhan and spread rapidly to all the nations worldwide, has led to 767 million cases and 6.9 million deaths globally [1]. The COVID pandemic has posed tremendous challenges to healthcare settings. Secondary bacterial infections in COVID-affected patients resulted in increased mortality and morbidity despite initiating appropriate antimicrobial therapy [2]. The most common presentation of secondary bacterial infections with COVID infection was bacterial pneumonia, followed by bacteremia and catheter-related sepsis [3].

Viral infections weaken the host immunity, paving the way for the development of secondary bacterial co-infection and superinfection [4,5]. This phenomenon was evident in influenza-related secondary bacterial infections, which contributed to severe illness and mortality during the epidemic and seasonal influenza outbreaks [5,6]. However, patients with secondary bacterial infections post-COVID infection were more severely ill, as reflected by disease severity markers, and had worse outcomes, reflected by a higher percentage of intubation or death than influenza patients [6]. Importantly, COVID-infected patients had more documented secondary bacterial infections than influenza patients, and the secondary bacterial infections were independently associated with death in COVID-infected patients but not in influenza patients [6].

The intensive use of antibiotics during the COVID pandemic led to the emergence of antibiotic resistance, which posed another challenge for the healthcare system as co-infection and superinfection with multidrug-resistant bacteria stretched healthcare units beyond their capabilities and resources [7,8]. The most common bacterial infections in patients with COVID infections were *Pseudomonas aeruginosa*, *Staphylococcus aureus*, *Streptococcus pneumoniae*, *Klebsiella pneumoniae*, *Haemophilus influenzae*, *Enterobacter* species and *Escherichia coli* [3,6,9]. The most frequent types of pathogens identified in the cultures of patients with secondary pneumonia were Gram-negative bacilli, followed by Gram-positive cocci, Gram-negative cocci and Gram-positive bacilli [3]. This case report was submitted as an abstract at the American Thoracic Society 2023 International Conference on May 23, 2023.

## Case presentation

An 81-year-old male patient with a history of hypertension and atrial fibrillation was diagnosed to have COVID infection and was hospitalized in a tertiary care hospital. The patient completed a 10-day course of dexamethasone and improved symptomatically and was discharged. However, post-discharge, the patient developed a productive cough, hemoptysis, palpitations, shortness of breath and left-sided chest pain over four days. The patient presented to the emergency department, where he was noted to be in rapid atrial fibrillation with a heart rate of 160 per minute. Chest x-ray revealed bilateral infiltrates in chest x-ray consistent with bilateral pneumonia. White cell count was elevated to 29,960/µL with a left shift. The patient was started on ceftriaxone and doxycycline for the management of bilateral pneumonia. The patient was started on amiodarone infusion and diltiazem. The patient was started on apixaban for anticoagulation. Rapid atrial fibrillation was resolved with amiodarone and diltiazem and reverted to normal sinus rhythm.

**Figure 1:**
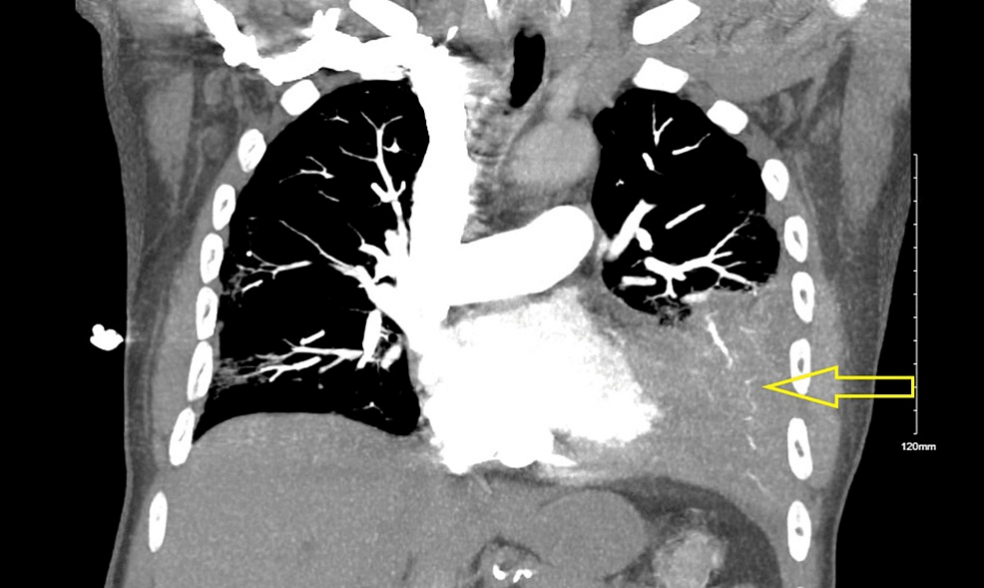
Computer tomogram pulmonary angiogram (CTPA) coronal section demonstrates partial consolidation and collapse of the left lower lobe as indicated by the arrow

**Figure 2:**
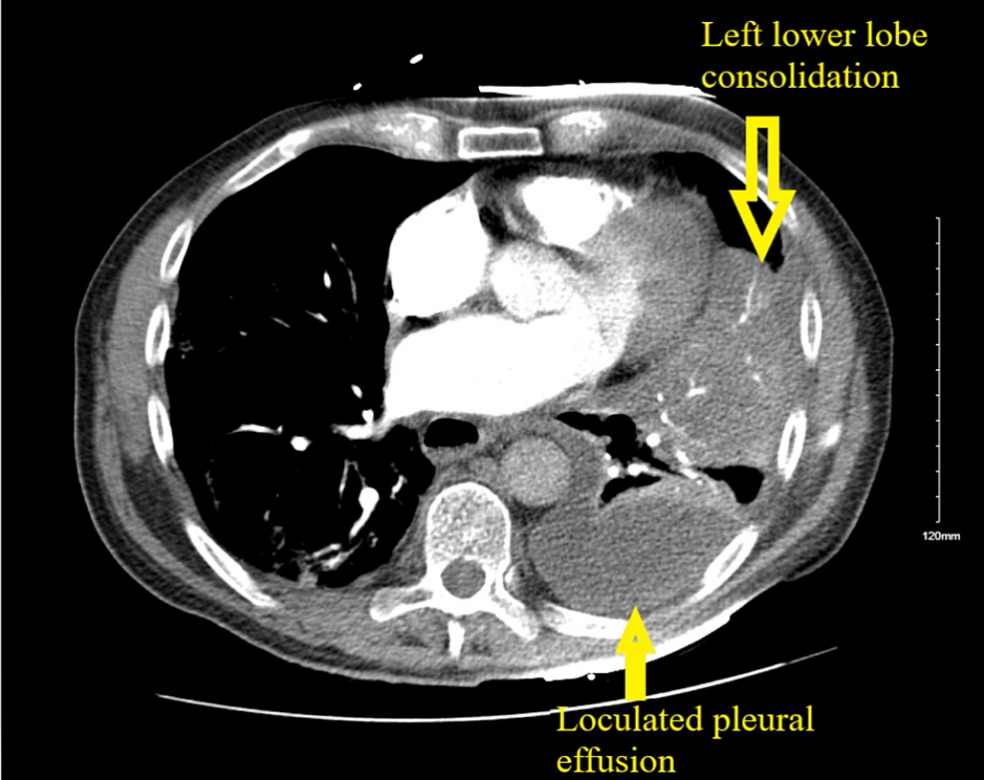
Computer tomogram pulmonary angiogram (CTPA) axial section demonstrates left-sided consolidation and left-sided pleural effusion as indicated by the arrows

Computer tomogram pulmonary angiogram (CTPA) revealed scattered peripheral ground glass infiltrates consistent with COVID infection and loculated left pleural effusion (Figures 1, 2). Sputum culture grew MRSA, and the patient was started on vancomycin. The patient underwent thoracocentesis, and pleural fluid analysis revealed empyema with a positive gram stain. Pleural fluid culture grew MRSA. The patient underwent left video-assisted thoracoscopic surgery (VATS) and was noted to have multiloculated left empyema with lung entrapment secondary to multiple perforated lung abscesses. Total decortication with parietal pleurectomy and tube thoracostomy was performed. However, post-VATS, the patient continued to be in respiratory distress with increasing oxygen requirements through the high-flow nasal cannula (HFNC). Repeat CT chest showed right-sided pleural effusion, worsening bilateral consolidation and vascular congestion. The patient had worsening MRSA pneumonia with persistent respiratory distress. The patient underwent right pleural drainage catheter placement, and 1 L of clear fluid was removed. The pleural fluid culture from right-sided pleural effusion was negative.

The patient developed severe acute renal failure with a serum creatinine of 4.8 mg/dL and blood urea nitrogen of 72 mg/dL and was started on intermittent hemodialysis. The patient developed multiorgan failure secondary to severe sepsis from worsening MRSA pneumonia. The patient was started on meropenem in addition to vancomycin because of worsening septic shock. The patient continued to deteriorate, became hemodynamically unstable and was started on vasopressor support. The patient developed severe acute respiratory distress syndrome (ARDS), resulting in acute hypoxic respiratory failure requiring intubation and mechanical ventilation. The patient was started on prone position ventilation for the management of severe ARDS.

The patient showed slow and gradual improvement in ARDS. After showing steady improvement in mechanical ventilation, gradual weaning from mechanical ventilation was started. The patient needed a protracted weaning from mechanical ventilation. The patient was extubated and started on non-invasive ventilation with bilevel-positive airway pressure (BiPAP) followed by HFNC. The sepsis resolved, and antibiotics were discontinued. After 15 days of intermittent hemodialysis, renal function gradually improved and hemodialysis was discontinued. The patient was gradually weaned from HFNC to a nasal cannula. After two months of stay in the ICU, the patient was severely deconditioned and was transferred to the medical ward, where the patient showed steady improvement with physiotherapy. The patient was discharged to the subacute rehab unit, where he had a complete physical recovery and was followed up in pulmonary and renal outpatient clinics.

## Discussion

Post-COVID pneumonia, secondary bacterial infections causing life-threatening complications were some of the biggest challenges during the COVID pandemic [2]. The patients suffering from secondary bacterial infections post-COVID pneumonia were at a higher risk of longer ICU and hospital stays and a higher risk of mortality and morbidity [9]. Researchers suggest approximately half of COVID-associated deaths can be attributed to such secondary infections [9]. Because of the longer ICU stay, the patients are subject to subsequent nosocomial infections and the emergence of multidrug-resistant organisms [9].

This case presentation illustrates the effect of severe nosocomial MRSA infection in an elderly post-COVID pneumonia patient who was recently admitted to a tertiary care center for the management of COVID pneumonia. The development of MRSA pneumonia immediately post-discharge after admission for COVID pneumonia was highly suggestive that the MRSA infection was nosocomial in nature [10,11]. The MRSA infection also fitted with the Centers for Disease Control and Prevention (CDC) definition of a superinfection as “an infection following a previous infection, especially when caused by microorganisms that are resistant or have become resistant to the antibiotics used earlier” [2]. Even though secondary bacterial infections were common post-COVID pneumonia, our case was unique in that the patient had a successful recovery from severe nosocomial MRSA pneumonia and empyema with life-threatening complications.

A major contributing factor in the development of secondary bacterial infections post-COVID pneumonia was COVID-associated immunosuppression resulting from platelet-driven systemic inflammation [2]. The immunosenescence from old age and significant comorbidities predisposed to severe secondary bacterial infection [2]. The patient was treated with a course of dexamethasone during his hospital stay, which played an important role in the treatment of cytokine release syndrome and the recovery from COVID pneumonia by its anti-inflammatory effect [12]. But steroids have been shown to predispose to secondary bacterial infection [12]. The immunosenescence, the steroid course and the COVID-associated immunosuppression led to severe MRSA pneumonia and empyema requiring thoracocentesis and urgent surgical intervention. Thus, the COVID-infected patients were at higher risk of developing secondary infections due to a combination of virus- and drug-induced immunosuppression [3,13].

In contrast to patients infected solely with COVID-19, co-infection with COVID-19 and *Staphylococcus aureus* demonstrates a higher patient mortality rate during hospital admission [13]. Clinical trials suggest that COVID-19 causes functional exhaustion of cluster of differentiation (CD)8 T cells and natural killer (NK) cells due to persistent stimulation from the virus, thus inducing T-cell exhaustion [14-16]. CD8 T cells secrete interferons (IFN-γ), perforin and granzymes to eradicate viruses, while CD4 helper T cells enhance CD8 cells and B cells to help them clear the viral pathogen [16]. The COVID-associated CD4 and CD8 T-cell functional exhaustion explains why our patient had such a severe MRSA superinfection with bilateral pneumonia and empyema resulting in life-threatening complications of severe septic shock, ARDS, severe acute renal failure and, ultimately, multiorgan failure [16,17].

## Conclusions

Most secondary bacterial infections post-COVID pneumonia occur as nosocomial infections due to exposure to multidrug-resistant organisms in healthcare facilities. Secondary bacterial infections lead to higher morbidity and mortality rate. This case illustrates the importance of early recognition of secondary bacterial infections post-COVID infection and the subsequent challenges in management, which necessitated the institution of a multidisciplinary team approach. A high index of suspicion for nosocomial MRSA infection and early initiation of medical management with appropriate antibiotics coupled with the timely surgical intervention of the MRSA empyema and ICU management of life-threatening complications resulted in the successful recovery of the patient.
